# Development and In Vitro Evaluation of 2-Methoxyestradiol Loaded Polymeric Micelles for Enhancing Anticancer Activities in Prostate Cancer

**DOI:** 10.3390/polym13060884

**Published:** 2021-03-13

**Authors:** Nabil A. Alhakamy, Osama A. A. Ahmed, Usama A. Fahmy, Shadab Md

**Affiliations:** 1 Department of Pharmaceutics, King Abdulaziz University, Jeddah 21589, Saudi Arabia; nalhakamy@kau.edu.sa (N.A.A.); osama712000@gmail.com (O.A.A.A.); usamafahmy@hotmail.com (U.A.F.); 2 Center of Excellence for Drug Research and Pharmaceutical Industries, King Abdulaziz University, Jeddah 21589, Saudi Arabia; 3 Mohamed Saeed Tamer Chair for Pharmaceutical Industries, King Abdulaziz University, Jeddah 21589, Saudi Arabia

**Keywords:** 2-methoxyestradiol, cell viability assay, cytotoxicity, optimization, polymeric micelles, prostate cancer

## Abstract

The present study aimed to formulate and optimize 2ME-loaded PMs (2ME-PMs) for enhancing the anticancer activity of 2ME in prostate cancer (PC). The 2ME-PMs were formulated using PEG-PLGA (PL), Tween 80 (TW80), and alpha-lipoic acid (ALA). The optimization was carried out using a Box-Behnken design with the PL, TW80, and ALA as the independent variables and particle size (PS) as the response. The formulation was optimized for the lowest possible PS, and the software suggested optimum formula with 100.282 mg, 2%, and 40 mg for PL, TW80, and ALA, respectively. The optimized PMs had spherical morphology with PS of 65.36 ± 2.2 nm, polydispersity index (PDI) of 0.273 ± 0.03, and entrapment efficiency of 65.23 ± 3.5%. The in vitro drug release was 76.3 ± 3.2% after 24 h. The cell line studies using PC-3 cells showed IC_50_ values of 18.75 and 54.41 µmol for 2ME-PM and 2ME, respectively. The estimation of tumor biomarkers was also carried out. The tumor biomarkers caspase-9 (17.38 ± 1.42 ng/mL), tumor protein P53 (p53) (1050.0 ± 40.9 pg/mL), nitric oxide (NO) (0.693 ± 0.03 pg/mL), interleukin-1β (IL-1β) (25.84 ± 2.23 pg/mL), nuclear factor kappa B (NF-kB) (0.719 ± 0.07 pg/mL), interleukin-6 (IL-6) (2.53 ± 0.16 folds), and cyclooxygenase-2 (COX-2) (3.04 ± 0.5 folds) were determined for 2ME-PMs and the results showed that these values changed significantly compared to those of 2ME. Overall, the results showed that the formulation of 2ME to 2ME-PMs enhances the anticancer effect. The exploration of the combined advantages of PEG, PLGA, ALA, and PMs in cancer therapy and the delivery of 2ME is the major importance of this research work. PEG reduces the elimination of 2ME, PLGA enhances 2ME loading, ALA has an inherent apoptotic effect, and PMs can efficiently target tumor cells.

## 1. Introduction

Prostate cancer (PC) represents a relevant epidemiological problem affecting men and is one of the most common types of cancer in men [1]. Chemotherapy has been instrumental in PC therapeutics and aiming at several drug targets has proved to improve therapeutic effectiveness [2]. There was a tremendous development in the approaches against PC. As a result, anti-androgen therapy emerged promising in terms of low toxicity but the clinical efficacy was not as expected [3]. Abiraterone and enzalutamide are such anti-androgen agents tried against PC. Among the chemotherapeutic agents, docetaxel has been useful to much extent in PC, but the toxicity limited its acceptability and use. Cabazitaxel was later introduced as a second-line chemotherapeutic agent. Meanwhile, immunotherapy and vaccine were also tried against PC [3].

As mentioned earlier, the initial efforts were to use anti-androgens against PC. However later, attention was focused on exploring the estrogen receptors as therapeutic targets in PC. Nevertheless, estrogen was found to cause PC and was even declared as a carcinogen [4]. As a result, selective estrogen receptor modulators and xenoestrogens emerged which were without any risk for cancer. Meanwhile, it was noticed that natural metabolites of estradiol are more beneficial than these agents. In such a pursuit, it was observed that 2-methoxyestradiol (2ME) have no affinity towards estrogen receptors, while inhibits angiogenesis [5]. Further, 2ME was proved to be useful in PC by Phase II clinical trials [6]. However, the bioavailability and anticancer effects of 2ME were not satisfactory on simple oral administration using conventional dosage forms such as tablets and capsules. Low aqueous solubility, a saturable absorption process, rapid metabolism, extensive biodistribution, and rapid elimination are some of the limiting factors which pose a barrier in developing a clinically successful formulation of 2ME [7,8,9]. Thus, to overcome such drawbacks in the cancer therapeutics with 2ME, a tailored 2ME delivery system is needed.

As always, nanotechnology and nanomedicine offered some prospects for 2ME. Formulation of nanocrystal dispersion of 2ME against PC was one among them [10]. Later, several nanoformulations including liposomes, polymeric nanoparticles, and inorganic nanoparticles were tried for the delivery of 2ME [11,12,13]. Each of these nanoformulations offered some advantages over conventional delivery systems. Interestingly, polyethylene glycol (PEG)-surface modified magnesium oxide nanoparticles decreased the viability of PC cell lines [13]. Meanwhile, the PEG-surface modified poly(lactic-co-glycolic acid) (PLGA) nanoparticles increased tumor accumulation [12]. These results suggested that PEGylation could be a good strategy for targeting PC. Further, the use of PEG-PLGA polymeric micelles (PMs) was described to have several advantages in cancer therapy [14]. In this type of PMs, the hydrophilic PEG chains of the co-polymer get directed towards the outside whereas the hydrophobic PLGA chains are directed towards the inside of the PMs [15]. This arrangement has distinct advantages of enhancing the blood circulation time by virtue of surface PEG chains and high drug loading of hydrophobic drugs by virtue of the hydrophobic PLGA chains in the core. Further, the PEG-PLGA copolymer possesses several advantages to suit drug delivery applications in the chemotherapy of PC [15]. Meanwhile, alpha-lipoic acid (ALA) can act both as a delivery system for an anticancer agent and an apoptotic agent. ALA increases the glutathione peroxidase activity and reduces oxidative stress resulting in tumor suppression and apoptosis [16].

In short, PEG reduces the elimination of 2ME, PLGA enhances 2ME loading, ALA has an inherent apoptotic effect, and PMs can efficiently target tumor cells. Thus, the novelty of the present work is that this study explores the combined advantages of PEG, PLGA, ALA, and PMs in cancer therapy. 

Thus, the present study aimed to enhance the cytotoxicity and therapeutic application of 2ME by formulation to PMs. Towards this aim, formulation, and evaluation of 2ME-loaded PEG-PLGA copolymer and ALAPMs (2ME-PMs) for therapeutic application in PC were carried out. The study also included optimization of the PM formulation through the design of experiments (DoE). The optimized 2ME-PMs were characterized in terms of particle size, zeta potential, and surface morphology. Finally, the optimized 2ME-PMs were evaluated for in vitro drug release, cytotoxicity, and tumor progression biomarkers.

## 2. Materials and Methods

### 2.1. Materials

2ME, PEG-PLGA copolymer (PLGA Mn: 11,500, PEG Mn: 2000), Tween 80, and alpha-lipoic acid were obtained from Sigma-Aldrich, St. Louis, MO, USA. Prostate cancer cell line types (PC-3 ATCC^®^ CRL-1435™) were gifted by department of Pharmacology, Faculty of Pharmacy, Al Azhar University, Cairo, Egypt. Further, all the reagents used in this article were analytic grade.

### 2.2. Formulation Development and Optimization of Polymeric Micelles 

#### 2.2.1. Design of Experiments

The optimization of the 2ME-PMs was done using a Box-Behnken design shown in Table 1. PEG-PLGA (PL), Tween 80 (TW80), and alpha-lipoic acid (ALA) were selected as the independent variables. Mean particle size (PS) was taken as the response. The design was generated and evaluated using Statgraphics software (Statgraphics Technologies, Inc., Warrenton, VA, USA).

#### 2.2.2. Formulation

Formulations of PMs were prepared. Briefly, different quantities of PEG–PLGA (100, 150, or 200 mg) and alpha-lipoic acid (ALA) (20, 30, or 40 mg) were dissolved in acetone (15 mL). 2ME (20 mg) was dissolved in the polymeric solution and then added to a 20 mL buffered solution (pH 9) containing Tween 80 (0.5, 1.25, or 2%). The resultant dispersion was stirred for 4 h to remove the organic solvent. 2ME-loaded PMs (2ME-PMs) were dialyzed to remove free drug using a cellulose dialysis membrane tube with a molecular weight cut-off range of 12,000–14,000. The dispersion was then lyophilized using the ALPHA 1-2/LD Plus freeze dryer (Martin Christ Gefriertrocknungsanlagen GmbH, Osterode am Harz, Germany).

#### 2.2.3. Optimization of 2ME-PMs

The optimization of PMs was carried out by the numerical method [17]. The minimum value for PS was set as the goal in the software. The optimum formula of 2ME-PMs as per the suggestion of the software was subjected to further studies.

### 2.3. Characterization of Drug-Loaded Polymeric Micelles

#### 2.3.1. Particle Size, Polydispersity Index, and Zeta Potential of Polymeric Micelles

The particle size (PS), polydispersity index (PDI), and zeta potential of the 2ME-PMs were determined using a Zetasizer Nano ZSP (Nano ZSP, Malvern, Worcestershire, UK). The PM samples were diluted 100 times in deionized water before measurements are carried out.

#### 2.3.2. Surface Morphology of Optimized Polymeric Micelles Using TEM

The transmission electron microscopy (TEM) analysis was performed after staining the PMs with phosphotungstic acid staining. The stained PMs were placed on a copper grid and examined under TEM (JEOL JEM-HR-2100, JEOL, Ltd., Tokyo, Japan).

#### 2.3.3. Entrapment Efficiency

For the determination of entrapment efficiency of the PMs, the specified weight of the freeze-dried PM was dissolved in chloroform. The mobile phase used was composed of 75:25 (*v*/*v*) methanol: water; flow rate, 1 mL/min; injection volume, 20 μL; sample retention time, 3.3 min. The samples were injected in HPLC Agilent 1260 Liquid chromatography with a diode-array detector (Agilent Technologies, Santa Clara, CA, USA) and analyzed for 2ME content using at 288 nm, as previously reported [18,19]. 2ME standard solution series was prepared in the range of 0.1–100 μg/mL, and HPLC analysis was performed to generate a standard curve. 2ME HPLC representative spectrum is available as a supplementary file (Figure S1 in supplementary materials).

### 2.4. In Vitro Drug Release Study

The in vitro drug release was carried out using a dialysis bag with a molecular weight cut-off of 12,000 Da. Briefly, the 2ME-PMs were introduced into the dialysis bag, tied, and immersed in the release medium. Phosphate-buffered saline (PBS) pH 6.8 (500 mL) containing Tween 80 (0.5%) as a solubilizer was used as the release medium in the study. The system was maintained at 37 °C in a shaker water bath throughout the study. The samples were withdrawn at time points of 2, 4, 6, 8, 10, 12, 18, 24, 32, 40, and 48 h and analyzed for 2ME content by HPLC.

### 2.5. In Vitro Cell Line Study in Prostate Cancer (PC-3 Cells)

#### 2.5.1. Cell Viability Using MTT Assay

The cell viability assay was carried out in PC-3 cell lines utilizing MMT assay. For the cell viability studies, cells were grown in 96-well plates to a density of 5 × 10^3^ cells/well and allowed to attach by incubating them overnight. The cells were then treated with 2ME-PMs corresponding to 2ME serial concentrations for 4 h at 37 °C. After removal of the supernatant, 100 μL of DMSO was employed for the solubilization of formazan crystals resulted from the treatment. The absorbance of the sample was measured at 570 nm using a microplate reader. In addition to the sample with 2ME-PMs, 2ME, placebo PMs and control (without any treatment) samples were also studied. The cell viabilities were then determined in triplicate and reported.

#### 2.5.2. Apoptotic Activity by Flow Cytometry

The apoptotic activity was measured using a previously reported method [20]. Briefly, PC-3 cells were incubated for 24 h with 2ME and 2ME-PM samples in a 6-well plate with a cell density of 1 × 10^5^ cells/well. A control sample with medium alone was also used. After treatment with the samples, the PC-3 cells were subjected to centrifugation and separation. Later, the cells were subjected to washing with phosphate-buffered saline and finally re-suspending in 500 μL of 1X binding buffer. The staining of the HCT cells was carried out using a commercially available kit (BD Bioscience, Franklin Lakes, NJ, USA). Staining was carried out using 5 μL each of Annexin V-FITC and propidium iodide (BDBioscience, CA, USA) at room temperature for 5 min in the dark. Flow cytometry (FACS Calibur, BD Bioscience, USA) was carried out and the data were analyzed (Multicycle software, Phoenix Flow Systems, San Diego, CA, USA) and reported.

#### 2.5.3. Cell Cycle Analysis by Flow Cytometry

Flow cytometry was performed for the cell cycle analysis. The procedure described for apoptotic activity was used for the cell cycle analysis also.

#### 2.5.4. Mitochondrial Membrane Potential Activity

The mitochondrial membrane potential was measured following a reported procedure with suitable modifications [21]. The experiment involved using an assay kit with tetramethylrhodamine methyl ester (TMRM) as the probe. The cells (1.5 × 10^4^ cells/well) were cultured in a 96-well plate. After 24 h, the cells were incubated with 2ME and 2ME-PM samples in DMEM medium (300 μL) supplemented with 10% FBS and 1% antibiotics. A control sample with DMEM medium alone was also maintained. Later, the medium was replaced with the probe solution and incubated in dark. Finally, the probe solution was replaced with the live-cell imaging buffer and analyzed using a flow cytometer.

#### 2.5.5. Estimation of Molecular Markers by ELISA Method

Caspase-9, tumor protein P53 (p53), nitric oxide (NO), tumor necrosis factor-alpha (TNF-α), interleukin-1β (IL-1β), nuclear factor kappa B (NF-kB), interleukin-6 (IL-6), and cyclooxygenase-2 (COX-2) were the biomarkers considered in the present study. The estimation of molecular markers was carried out using ELISA kits available for each type of biomarker (Invitrogen^®^, Thermo Fisher Scientific, Waltham, MA, USA). Briefly, PC-3 cells (5 × 10^4^ cells/well) were seeded in a 96-well plate and incubated. The cells were treated with 2ME and 2ME-PM samples. A control without any sample treatment was also carried out. All the samples were allowed to equilibrate at room temperature. Later, 100 μL of the reagent was added to each well containing 100 μL of the medium. After mixing for 30 s at 500 rpm, the samples were kept aside at room temperature for 30 min. Finally, caspase-9, p53, NO, TNF-α, IL-1β, NF-kB, IL-6, and COX-2 were estimated using the corresponding ELISA kits following the protocol suggested by the manufacturer.

#### 2.5.6. Effect of 2ME-PMs on Bax and Bcl-2 Using RT-PCR

Bcl-2 and Bax protein expressions were analyzed in all samples by RT-PCR [22]. The Qiagen RNA extraction/BioRad SYBER green PCR MMX kit was used in the study. A Rotorgene RT- PCR system was used for reading. The system was equipped with Rotor-Gene 1.7.87 software. The sequences of the primers used for the study are shown in Table 2.

### 2.6. Statistical Analysis

The experiments were carried out in triplicate and the results are presented as mean ± standard deviation. The statistical significance was examined using one-way ANOVA followed by Tukey multiple comparison test and *p*-value < 0.05 was considered significant.

## 3. Results and Discussion

### 3.1. Formulation Development and Optimization of Polymeric Micelles

#### 3.1.1. Design of Experiments

The 2ME-PMs were prepared with PL, TW80, and ALA as independent factors for optimization. Fifteen formulations suggested by the software were prepared and evaluated for PS. The data obtained for PS for these 15 formulations are given in Table 3.

The analysis of variance data obtained for the PS is presented in Table 4. From the P-values, it was found that all the independent factors selected for the study (PL, TW80, and ALA) were statistically significant in their influence on the mean PS of 2ME-PMs. Besides, the interaction term between PL and ALA was also found to be significant. Further, among these independent factors, PL had the most significant influence. The R-squared value and the adjusted R-squared values were 96.9621 and 91.4939%, respectively. Further, the observed and fitted values for the PS were in good agreement with each other (Table 3). 

The polynomial equation suggested by the software for PS is provided in Equation (1). The regression coefficients in the polynomial equation could be used to identify the effect of each independent factor on the response. A negative sign for the regression coefficient for any factor suggests a negative effect of that factor on PS and vice versa. Thus, a regression coefficient value of +300.5 for PL suggests that higher PL increases the particle size. Meanwhile, regression coefficient values of −72.4444 and −64.2222 for TW80 and ALA, respectively, showed that higher values of both these independent factors contribute to a decrease in the PS. The magnitude of the regression coefficients can be compared to reach an estimation of the extent of the effect of the independent factors on the PS. Thus, PL with the highest magnitude of 300.5 for regression coefficient was observed to have a higher influence than TW80 and ALA on PS. Meanwhile, the significant interaction term ‘C’ between PL and ALA has a regression coefficient value of +124.0. This can contribute a reverse of the individual effect of ALA on PS, and produces higher PS at higher ALA quantities.

PS = −66.75 + 300.5A − 72.4444B − 64.2222C − 88.0A^2^ − 8.0AB + 124.0AC + 40.8889B^2^ − 62.2222BC + 10.6667C^2^ (1)

**Figure 1:**
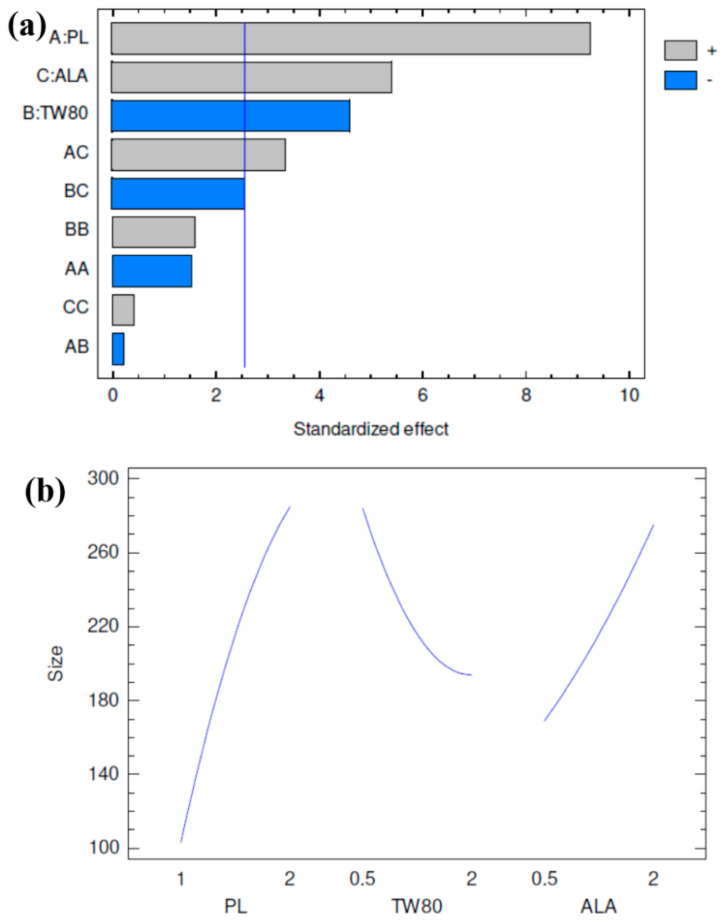

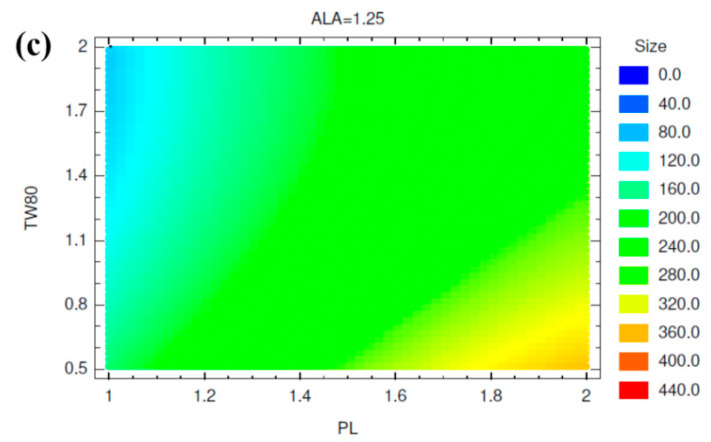
Results for particle size (PS) after carrying out design of experiments by Box-Behnken design for the formulation of 2-methoxyestradiol-loaded polymeric micelles (2ME-PMs) prepared with PEG-PLGA (PL), Tween 80 (TW80), and alpha-lipoic acid (ALA) as independent factors (**a**) standardized Pareto chart (**b**) main effects plot (**c**) contours of the response surface.

The Pareto chart is shown in Figure 1a and it suggested significant effects of PL, ALA, TW80 and interaction effect of PL and ALA on PS. It is well established that increasing the polymer weight increases the size of PMs [23]. Thus, both PL and ALA, which form the components of the PMs can increase particle size when used in higher quantities. Meanwhile, it was noted that the higher TW80 (surfactant) concentration decreased PS. However, this result was opposite to those of reported studies where higher surfactant concentration increased the PS of PMs [24]. Nevertheless, in general, higher surfactant concentration generally results in lower particle size of nanostructures [25]. 

The main effects plot for PS (Figure 1b) also confirmed the results of the Pareto chart. The plot clearly shows that PL increases the PS significantly. Meanwhile, ALA also increased the PS, but to a lesser extent than PL. TW80 concentration was found to decrease the PS with a lower effect towards its higher concentration selected for the study. The contours of the estimated response surface are shown in Figure 1c. From the contours, it can be confirmed that higher values of PL and lower values of TW80 lead to the highest PS when the value of ALA is kept at a fixed level. Previous reports have shown the utilization of PEG-PLGA for the preparation of PMs [26,27,28]. The inclusion of TW80 and ALA is thought to stabilize the formation of polymeric micelles. ALA has been reported to form micelles with Vitamin E TPGS [29].

#### 3.1.2. Optimization of 2ME-PMs

The optimum formulation suggested by the software is shown in Table 5. The optimum PS suggested by the software was 62.76 nm.

### 3.2. Characterization of Drug-Loaded Polymeric Micelles

#### 3.2.1. Particle Size, Polydispersity Index, and Zeta Potential of Polymeric Micelles

The particle size of the optimized 2ME-PMs was found to be 65.36 ± 2.2 nm. This particle size was well in the range of 10–200 nm suggested for tumor-targeted delivery [30]. From the observed particle size of around 65 nm, it can be expected that the internalization of the 2ME-PMs could occur via clathrin-mediated endocytosis [31]. Meanwhile, the PDI value of the optimized 2ME-PMs was found to be acceptable with 0.273 ± 0.03. A PDI value of less than 0.5 indicates narrow size distribution [32]. A still lower value of 0.3 is suggested for nanostructures [33]. Thus, in the present study, a PDI value of less than 0.3 indicated a highly monodisperse system for the obtained 2ME-PMs. The zeta potential of optimized blank PMs and 2ME loaded PMs were −24.9 ± 2.09 mV and −32.8 ± 3.15 mV, respectively, which indicate the stability of prepared polymeric micelles.

#### 3.2.2. Surface Morphology of Optimized Polymeric Micelles Using TEM

**Figure 2:**
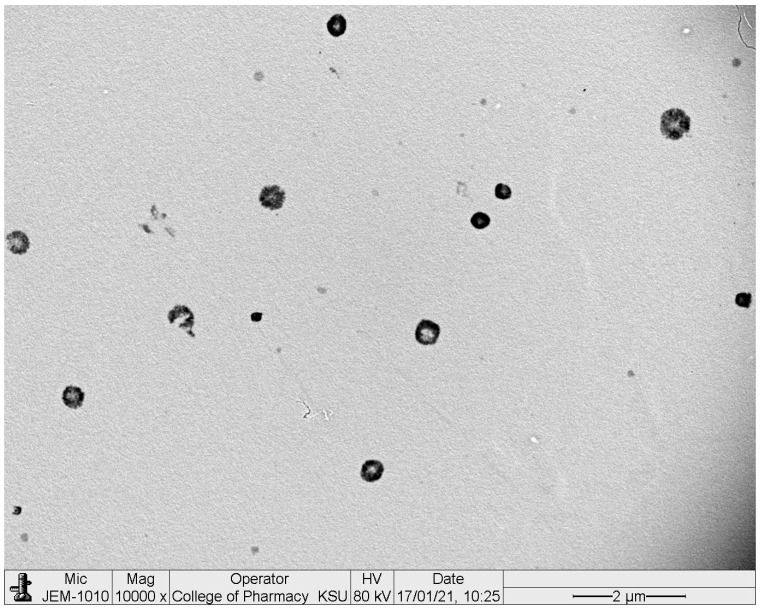
TEM image of optimized 2-methoxyestradiol-loaded polymeric micelles (2ME-PMs) prepared with the optimum formula of 100.282 mg, 2%, and 40 mg for PEG-PLGA (PL), Tween 80 (TW80), and alpha-lipoic acid (ALA), respectively.

In the TEM image (Figure 2), the surface morphology of the optimized polymeric micelles was found to be similar to the reported system of PLGA PMs [34]. Spherical PMs without any aggregation are seen in the TEM image. The appearance of irregular/rough surfaces is due to the protrusions of the PEG chains which are hydrophilic in nature [15].

#### 3.2.3. Entrapment Efficiency

The entrapment efficiency of 2ME in PEG-PLGA copolymer PMs was found to be 65.23 ± 3.5%. Interestingly, this value was higher than that observed for a peptide drug in similar PMs, where it was only around 55% [35]. In the case of PEG-PLGA PMs, the entrapment of the drug occurs mostly in the core formed by the PLGA polymer [15]. This might have contributed to the higher entrapment efficiency of PEG-PLGA PMs towards 2ME. This is also evident from the results of a study with PLGA nanoparticles of 2ME, where it shows that higher PLGA content causes enhancement of 2ME entrapment efficiency [12].

### 3.3. In Vitro Drug Release Study

**Figure 3:**
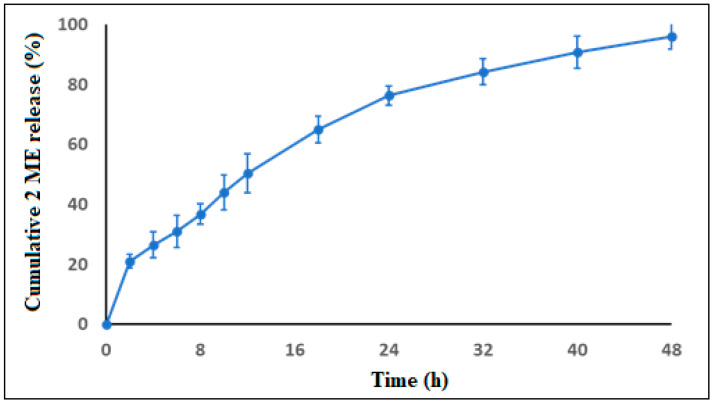
In vitro release of 2-methoxyestradiol (2ME) from 2ME-loaded polymeric micelles (2ME-PMs) in phosphate-buffered saline (PBS) at 37 °C, pH 6.8 (500 mL) containing Tween 80 (0.5%) as a solubilizer by dialysis bag method.

The in vitro release profile of 2ME from the 2ME-PMs in pH 6.8 phosphate buffer is shown in Figure 3. The release of 2ME was sufficiently sustained for 48 h. A slight burst release was observed with 21.0 ± 2.3% at 2 h. Later, the 2ME release was sustained well and reached a value of 76.3 ± 3.2% and 96.1 ± 4.3% after 24 and 48 h, respectively. Thus, it indicated that the complete drug release takes more than 24 h. Thus, the polymeric micelles formed of PEG-PLGA block copolymer were able to provide a platform that could sustain 2ME for more than 24 h. Entrapment of the hydrophobic 2ME into the core of the PMs composed of PLGA could be attributed to such a strong drug-sustaining behavior [15]. Further, these observations were in concurrence with previous reports on PEG-PLGA based PMs [27].

### 3.4. In Vitro Cell Line Study in Prostate Cancer (PC-3 Cells)

#### 3.4.1. Cell Viability Using MTT Assay

**Figure 4:**
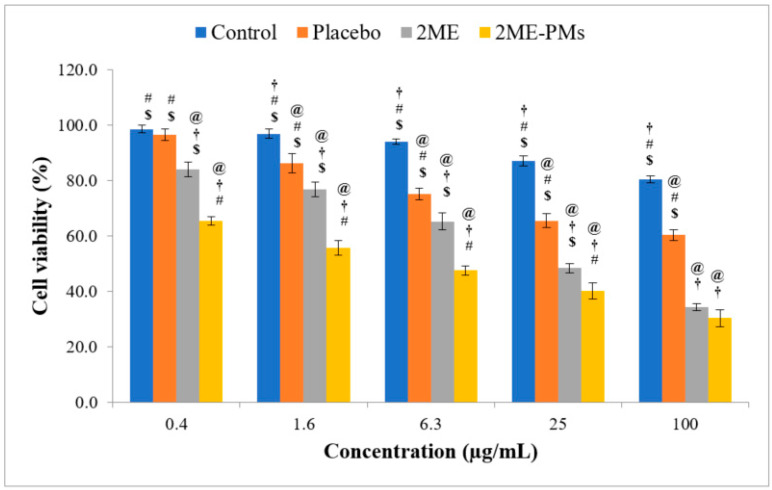
Cell viability assay results of control, placebo, 2-methoxyestradiol (2ME), and 2ME-loaded polymeric micelles (2ME-PMs) following cell viability assay using 3-(4,5-dimethylthiazol-2-yl)-2,5-diphenyl tetrazolium bromide (MTT) in PC-3 cells [Statistical inferences: @, *p* < 0.05, compared with control; †, *p* < 0.05, compared with placebo; #, *p* < 0.05, compared with 2ME; $, *p* < 0.05, compared with 2ME-PMs].

The MTT assay was performed in PC-3 cells to check whether the formulation of 2ME to 2ME-PMs enhances the cytotoxicity or not. From the results (Figure 4), it is clear that higher concentrations of 2ME and 2ME-PMs show significantly higher cytotoxicity. Among them, the cell viability was much less for 2ME-PMs compared to the pure drug. Such enhanced cytotoxicity of drug-loaded PEG-PLGA micelles than pure drug has been demonstrated in a previous study with doxorubicin [27]. Enhanced uptake of drug-loaded PEG-PLGA micelles relative to the free drug was attributed to this effect. Thus, the uptake of 2ME-PMs by PC3 cells could be better than 2ME to show such a notable effect. 

In addition, the placebo PMs also showed a slight increase in cytotoxicity at higher concentrations. This signifies that the placebo PMs also have cytotoxic effects to some extent at higher concentrations. It is important to note that the concentrations mentioned in the cell viability assay are of 2ME. Based on the entrapment efficiency of around 65% observed in the present study, 100 µg/mL of 2ME corresponds to approximately 1170 µg/mL of placebo PMs. This placebo concentration in turn comprises 310 and 750 µg/mL of ALA and PL, respectively. These high concentrations of formulation excipients might have contributed to the slight decrease in the cell viability at higher studied concentrations. The concentration-dependent increase in cytotoxicity of PEG-PLGA polymer has been demonstrated in previous studies, even at concentrations below 200 µg/mL [36]. Further, it has been demonstrated that even the shape of nanoparticles formed with PEG-PLGA polymer influences their cytotoxicity, and shows cytotoxicity even below a concentration of 100 µg/mL [37]. Interestingly, this reported study by Zhang et al. confirmed that needle-shaped nanoparticles formed with PEG-PLGA polymer are more cytotoxic than their spherical-shaped nanoparticles, and even at a low concentration of 10 µg/mL. These results have high relevance to the present study results with placebo PMs. PMs can be considered imperfectly spherical, and with protrusions resembling needle-like structures. Thus, cytotoxicity higher than spherical and lower than needle-like nanostructures can be expected from PEG-PLGA PMs. In the case of the influence of ALA, a study conducted by Feuerecker et al. with ALA concentrations in the range of about 500–2000 µg/mL has shown that ALA decreases cell viability of cancer cells [38]. From the detailed examination of the results presented in this reported study by Feuerecker et al., it is reasonable to propose that ALA concentrations even below 500 µg/mL can have a cytotoxic effect. Therefore, an ALA concentration of around 310 µg/mL in the placebo PMs could also have contributed to the reduction of cell viability by placebo PMs in our study. Furthermore, ALA can also show time-dependent toxicity and is established in a previous study [39]. Therefore, based on the established effects of PL and ALA present in placebo PMs, a concentration-dependent lowering of cell viability by placebo PMs in the present study could be justified. Moreover, such an effect of placebo PMs is observed in previous reports also [40]. 

Meanwhile, the IC_50_ values were found to be 18.75 and 54.41 µmol for 2ME-PM and 2ME, respectively. This also confirmed the higher cytotoxicity of 2ME-PMs than the pure drug. However, at the highest studied concentration of 100 µg/mL, there was no significant difference between cell viabilities produced by 2ME and 2ME-PMs. This result indicated that PMs are able to enhance the cytotoxicity of 2MEs significantly at low drug concentrations. In summary, it was confirmed that the formulation of 2ME to polymeric micelles enhances its cytotoxicity. Better cellular internalization of PMs could be attributed to this observation [41].

#### 3.4.2. Apoptotic Activity by Flow Cytometry

**Figure 5:**
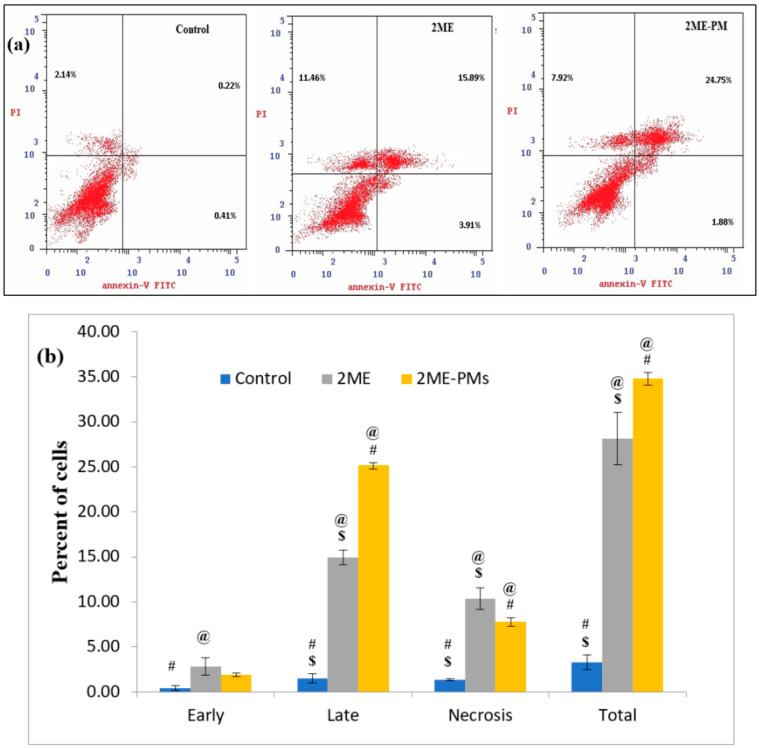
Apoptotic activity of samples by Annexin V-FITC and propidium iodide and flow cytometry (**a**) Dot plots showing PC-3 cells treated with control, 2-methoxyestradiol (2ME), and 2ME-loaded polymeric micelles (2ME-PMs) (**b**) Bar diagram showing a quantitative data of percent of cells in various stages of apoptosis. [Statistical inferences: @, *p* < 0.05, compared with control; #, *p* < 0.05, compared with 2ME; $, *p* < 0.05, compared with 2ME-PMs].

The apoptotic activities of the samples are shown in Figure 5. From the results, it can be seen that the 2ME-PMs significantly increased the late and total apoptotic cell percentages. This indicated that the formulation of 2ME to 2ME-PMs enhances the apoptotic activity of 2ME. The percentage of cells was significantly higher for 2ME-PMs than that for 2ME in the late apoptotic phase than in any other phase. Meanwhile, 2ME alone produced a significantly higher cell percentage in the early necrosis phase. In the early apoptotic phase, there was no significant difference between the percent of cells for 2ME to 2ME-PMs.

#### 3.4.3. Cell Cycle Analysis by Flow Cytometry

**Figure 6:**
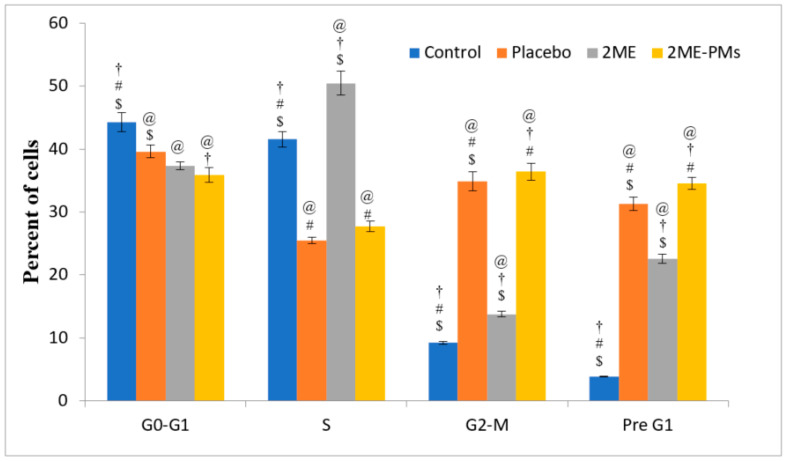
Histograms derived from cell cycle analysis of samples by Annexin V-FITC and propidium iodide and flow cytometry. The percent of cells observed in each phase was quantified after control, placebo, 2-methoxyestradiol (2ME), and 2ME-loaded polymeric micelle (2ME-PM) treatments in PC-3 cells. [Statistical inferences: @, *p* < 0.05, compared with control; †, *p* < 0.05, compared with placebo; #, *p* < 0.05, compared with 2ME; $, *p* < 0.05, compared with 2ME-PMs].

The results of cell cycle analysis (Figure 6) showed that the untreated control cells show good proliferation with a high percentage of cells in the G0-G1 and S phases. The percent of cells in the G2-M and Pre G1 phases can be used as a measure of the apoptotic ability of anticancer agents. The results showed that 2ME-PMs caused a significantly higher percentage of cells in the G2-M and Pre G1 phases compared to 2ME (Figure 4). This confirmed a higher cytotoxic effect of 2ME-PMs than 2ME. Surprisingly, the placebo PMs also showed significant cell percent in the G2-M and Pre G1 phases. This observation indicated that the placebo PMs themselves can have cytotoxicity. In general, placebo PMs do not show inherent cytotoxicity [42]. Nevertheless, it can happen depending on the polymer employed for the fabrication of PMs. Besides, there was no significant difference in the percent of cells in the G2-M phase by placebo PMs and 2ME-PMs. Meanwhile, a slightly higher increase in the percent of cells is noted for 2ME-PMs in Pre G1 phases compared to placebo PMs.

#### 3.4.4. Mitochondrial Membrane Potential Activity

**Figure 7:**
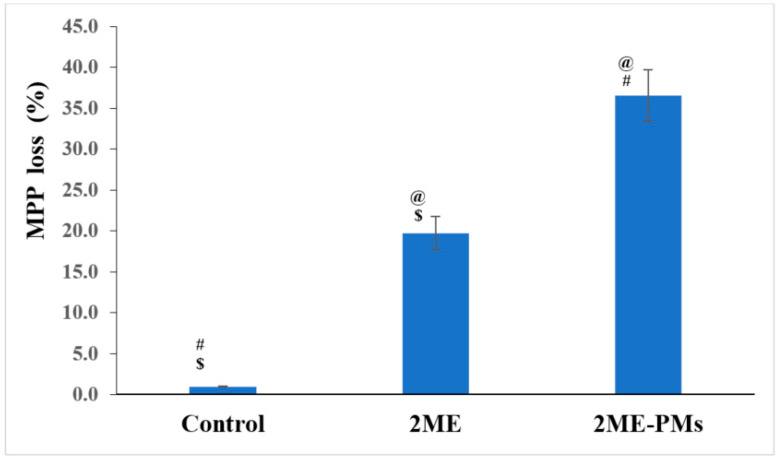
Mitochondrial membrane potential (MMP) activities of control, 2-methoxyestradiol (2ME), and 2ME-loaded polymeric micelles (2ME-PMs) in PC-3 cells determined using tetramethylrhodamine methyl ester probe and flow cytometry. The histograms represent the percentage loss of MPP after treatment with the samples. [Statistical inferences: @, *p* < 0.05, compared with control; #, *p* < 0.05, compared with 2ME; $, *p* < 0.05, compared with 2ME-PMs].

Cellular dysfunction or apoptosis leads to loss of integrity of the mitochondrial membrane [43]. This loss of integrity can be assessed by changes in MMP mitochondrial. A high MMP loss of 36.6 ± 3.2% was observed for 2ME-PMs compared to 2ME with an MMP loss of 19.7 ± 2.0% (Figure 7). Thus, it was inferred that the PMs can enhance the loss of MMP significantly. The loss of MMP indicates the first stage of apoptosis and thus, 2ME-PMs facilitate the apoptosis of cancer cells far better than the pure drug. Similar results are reported for nanostructured formulations of drugs [44].

#### 3.4.5. Estimation of Molecular Markers by ELISA Method

The present study estimated various molecular markers produced by PC-3 cell lines by ELISA methods. These studies were aimed to provide an outlook of the effect of encapsulation of 2ME into PEG-PLGA PMs in the production of these biomarkers. Caspase-9, p53, NO, TNF-α, IL-1β, NF-kB, IL-6, and COX-2 were determined for this purpose in the present study. 

**Figure 8:**
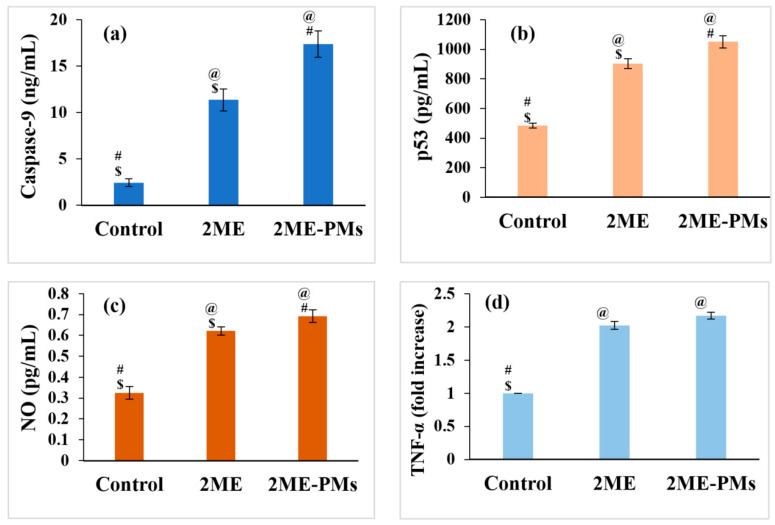

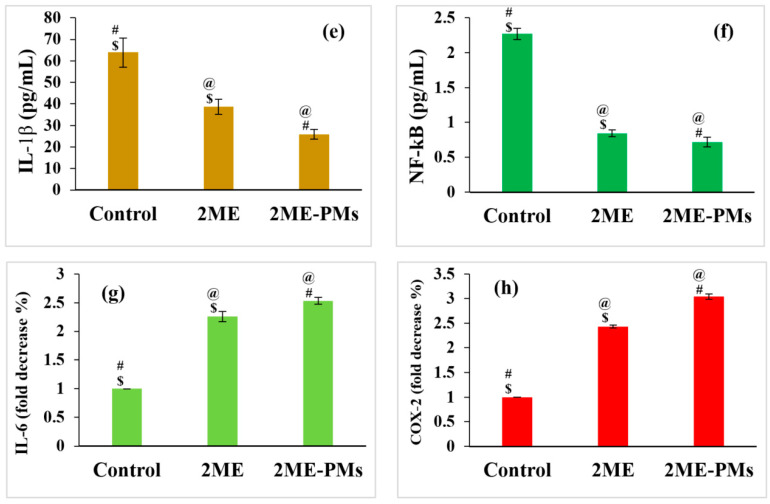
Effect of 2-methoxyestradiol (2ME) and 2ME-loaded polymeric micelles (2ME-PMs) on various biomarkers (**a**) Caspase-9 (**b**) tumor protein P53 (p53) (**c**) nitric oxide (NO) (**d**) tumor necrosis factor-alpha (TNF-α) (**e**) interleukin-1β (IL-1β) (**f**) nuclear factor kappa B (NF-kB) (**g**) interleukin-6 (IL-6) (**h**) cyclooxygenase-2 (COX-2) [Statistical inferences: @, *p* < 0.05, compared with control; #, *p* < 0.05, compared with 2ME; $, *p* < 0.05, compared with 2ME-PMs].

Caspase-9 is activated in the early apoptotic phase of cells [45]. Thus, a measure of the production of caspase-9 will provide an assessment also for the therapeutic potential of cytotoxic agents. In the present study, a caspase-9 assay was carried out to know whether the formulation of 2ME to 2ME-PMs has a significant effect on apoptosis. The results were as expected with a significantly high caspase-9 production by the 2ME-PMs compared to 2ME (Figure 8a). The caspase-9 content was 17.38 ± 1.42 and 11.35 ± 1.17 ng/mL for 2ME-PMs and 2ME, respectively. Both these values were significantly higher than that observed for the control (2.42 ± 0.40 ng/mL).

p53 is a transcription factor that activates a gene responsible for apoptosis. In simple words, a higher value of p53 assay could imply high cytotoxicity. 2ME-PMs were found to produce significantly higher levels of p53 compared to both control and 2ME samples (Figure 8b). The p53 concentration was found to be 1050.0 ± 40.9 pg/mL for 2ME-PMs. Meanwhile, the NO content was found to be in the order control < 2ME < 2ME-PMs (Figure 8c). Here, there was a significant difference between the NO level produced by 2ME-PMs and 2ME with a higher value of 0.693 ± 0.03 pg/mL for 2ME-PMs. 

The result TNF-α was not favorable for 2ME-PMs as it indicated no significant difference from the TNF-α level for 2ME (Figure 8d). TNF-α is a cytokine for cell signaling and induces apoptosis. Thus, its higher level can indicate apoptosis. In the present study, both 2ME and 2ME-PMs have a similar effect of significantly high TNF-α level than the control. However, the formulation of 2ME to 2ME-PMs was not found to influence the TNF-α production. TNF-α is produced mainly by macrophages [46]. It is well established that the surface modification of nanoparticles with PEG reduces interaction with macrophages and hence avoids nanoparticle uptake [47]. Thus, the presence of PEG chains towards the outer surface part of the PMs can reduce the interactions with macrophages. This might have contributed to the unresponsive behavior of 2ME-PMs in TNF-α level compared to 2ME.

IL-1β promotes tumor growth and progression [48]. Further, it is reported that NF-kB regulates IL-1β transcription [49]. Thus, lower levels of NF-kB and IL-1β could be taken as a measure of inhibition of tumor growth and progression. The results of the present study were in line with this expectation. The study results showed that 2ME-PMs significantly reduced both IL-1β (Figure 8e) and NF-kB (Figure 8f) levels compared to the control and 2ME. The concentrations of IL-1β and NF-kB were 25.84 ± 2.23 and 0.719 ± 0.07 pg/mL, respectively, for 2ME-PMs.

IL-6 level was also determined after treatment with 2ME and 2ME-PM samples. Interestingly, a low level of IL-6 potentiates the cytotoxicity of TNF-α [50]. Thus, treatments that can lower or decrease the IL-6 level can be assumed to contribute to cytotoxicity. The results of IL-6 levels in the present study (Figure 8g) showed a high reduction of IL-6 levels by both 2ME and 2ME-PM samples, compared to the control. Meanwhile, the reduction of IL-6 level by 2ME-PMs was significantly more than the reduction caused by the 2ME.

COX-2 can promote tumor growth and cause suppression of tumor immunity. Thus, COX-2 could be considered as a biomarker in cancer immunotherapy [51]. Like IL-6 levels, both the 2ME and 2ME-PM samples caused a significant decrease in the COX-2 levels compared to the control (Figure 8h). Thus, both 2ME and 2ME-PMs demonstrated significant cytotoxic effects. Furthermore, there was a significant reduction in COX-2 level by 2ME-PM compared to 2ME; indicated a higher cytotoxic effect for the PM formulation compared to the pure drug.

#### 3.4.6. Effect of 2ME-PMs on Bax and Bcl-2 Using RT-PCR

**Figure 9:**
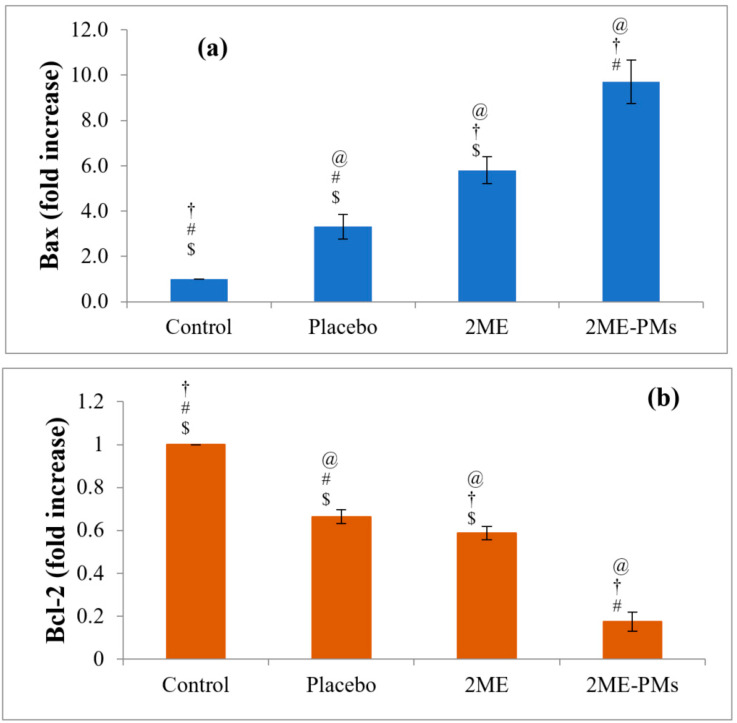
Bax (**a**) and Bcl-2 (**b**) gene expressions after treatment with control, placebo, 2-methoxyestradiol (2ME), and 2ME-loaded polymeric micelles (2ME-PMs). [Statistical inferences: @, *p* < 0.05, compared with control; †, *p* < 0.05, compared with placebo; #, *p* < 0.05, compared with 2ME; $, *p* < 0.05, compared with 2ME-PMs].

In simple words, Bax is an apoptosis promoter and Bcl-2 is an apoptosis inhibitor. Thus, determining the expression levels of Bax and Bcl-2 would be beneficial in the assessment of the ability of any system to cause apoptosis. A high level of expression of Bax genes indicates that the system causes apoptosis [52]. Here, the results showed that the highest increase in Bax expression was for 2ME-PMEs with a 9.7 ± 0.95 folds increase compared to the control (Figure 9a). This value was significantly higher than that for 2ME with a value of 5.8 ± 0.59 folds. The placebo PMs also showed a slight increase in the Bax gene expression. This result was in agreement with the cell viability studies also.

In the case of Bcl-2 gene expression, a reverse order to the effect of Bax gene expression was noted for all the samples. This is acceptable as the Bcl-2 gene expression shows apoptosis inhibition, a process reverse to Bax gene expression. Thus, substances that cause apoptosis and show cytotoxicity would have low values for the Bcl-2 gene expression. In the present study, the lowest Bcl-2 expression was noted for 2ME-PMEs with a value of 0.17 ± 0.05 folds only when compared to the control and 2ME-PMEs (Figure 9b). The 2ME sample showed a significantly low value of 0.59 ± 0.03 folds compared to control.

## 4. Practical Applications, Future Research Perspectives, and Challenges

The results of the present study have significant practical applications. The selection of PL, TW80, and ALA had a significant influence in enhancing the cytotoxicity of 2ME. Moreover, the very low PS of 2ME-PMs favor accumulation in tumor cells by enhanced permeability and retention effect. Thus, the developed 2ME-PMs have been able to provide better cytotoxicity than 2ME alone. Tailoring of the 2ME-PMs can be done for improvement in its therapeutic application. The inclusion of a stimuli-responsive moiety/approach in the 2ME-PMs could further enhance its anti-cancer effect. The versatility in the polymer systems provides a plethora of opportunities for PM-based cancer therapy. The fine-tuning of the core and corona of the PMs is highly achievable by the selection of appropriate co-polymers. This can provide desired encapsulation efficiency, drug release, and tumor-targeting and retention. Therefore, increasing the drug loading efficiency and simplifying the preparation method to suit for scale-up can be taken as future research objectives. Moreover, optimum drug loading and restricting loaded drugs in the PMs from releasing into the circulatory system before reaching the target tumor cells have to be achieved. Additionally, PMs have to effectively address the pathophysiological barriers in delivering 2ME to the target site. The possibility of scale-up of the 2ME-PMs is highly possible as evidenced by the successful scale-up of the manufacturing process of similar systems for pharmaceutical applications [53]. Presently, lower encapsulation efficiency and the batch process employed in the preparation of 2ME-PMs are the possible limitations to use this methodology for commercial application. Importantly, establishing a continuous process/method of preparation is very essential for the manufacture of 2ME-PMs for commercial application. Further, assessing the stability of 2ME-PMs is also required before it can be commercialized for clinical use. 

## 5. Conclusions

In summary, 2ME-PMs were prepared using PL, TW80, and ALA. The optimized PMs were satisfactory in terms of PS (65.36 ± 2.2 nm), PDI (0.273 ± 0.03), surface morphology, and entrapment efficiency (65.23 ± 3.5%). The sustained in vitro drug release showed a burst release of 21.0 ± 2.3% at 2 h and reached the highest value of 76.3 ± 3.2% after 24 h. The tumor biomarkers caspase-9, p53, NO, IL-1β, NF-kB, IL-6, and COX-2 were significantly changed by 2ME-PMs compared to 2ME. Meanwhile, both the 2ME and 2ME-PMs had similar effects on TNF-α, but significant changes compared to the control without any treatment. Overall, 2ME-PMs were successfully developed and optimized for enhancing the anticancer activity of 2ME. The enhancement of the anticancer activity of 2ME by formulation to 2ME-PMs could be considered as the important and specific output of the present study. The formulation of 2ME-PMs could significantly change the apoptotic biomarkers or parameters compared to 2ME alone. Thus, it is possible to formulate 2ME as PMs, and with enhanced cytotoxic effects. 

## Figures and Tables

**Table 1:** Experiment trials with codes and values for carrying out design of experiments by Box-Behnken design for the formulation of 2-methoxyestradiol-loaded polymeric micelles (2ME-PMs) using PEG-PLGA (PL), Tween 80 (TW80), and alpha-lipoic acid (ALA) as independent factors.

Run	Factor Codes			Factor Values		
	Factor-PEG-PLGA (PL)	Factor-Tween 80 (TW80)	Factor-Alpha-lipoic Acid (ALA)	Factor-PL (mg)	Factor-TW80 (%)	Factor-ALA (mg)
1	0	0	0	150	1.25	30
2	1	0	1	200	1.25	40
3	1	−1	0	200	0.5	30
4	−1	1	0	100	2	30
5	0	−1	1	150	0.5	40
6	−1	0	−1	100	1.25	20
7	−1	−1	0	100	0.5	30
8	1	0	−1	200	1.25	20
9	0	0	0	150	1.25	30
10	1	1	0	200	2	30
11	0	0	0	150	1.25	30
12	−1	0	1	100	1.25	40
13	0	1	−1	150	2	20
14	0	−1	−1	150	0.5	20
15	0	1	1	150	2	40

**Table 2:** Sequences of the primers used in real-time polymerase chain reaction (RT-PCR) assays for studying the effect of 2-methoxyestradiol-loaded polymeric micelles (2ME-PMs) on Bcl-2 and Bax protein expressions.

Bax F	5’-TGGCAGCTGACATGTTTTCTGAC-3’
Bax R	5’-TCACCCAACCACCCTGGTCTT-3’
Bcl-2 F	5’-TCGCCCTGTGGATGACTGA-3’
Bcl-2 R	5’-CAGAGACAGCCAGGAGAAATCA-3’
GAPDH F	5’-AATGCATCCTGCACCACCAA-3’
GAPDH R	5’-GATGCCATATTCATTGTCATA-3’

**Table 3:** The observed and fitted values of particle size (PS) obtained in various trials by Box-Behnken experimental design for the formulation of 2-methoxyestradiol-loaded polymeric micelles (2ME-PMs) using PEG-PLGA (PL), Tween 80 (TW80), and alpha-lipoic acid (ALA) as independent factors.

Run	Independent Factors			Dependent Factor	
	Factor-PEG-PLGA (PL) (mg)	Factor-Tween 80 (TW80) (%)	Factor-Alpha-lipoic Acid (ALA) (mg)	Mean Particle Size (PS) (nm)	
				Observed Values	Fitted Values
1	150	1.25	30	216	216.0
2	200	1.25	40	226	202.0
3	200	0.5	30	194	218.0
4	100	2	30	214	216.0
5	150	0.5	40	181	191.25
6	100	1.25	20	98	84.25
7	100	0.5	30	342	355.75
8	200	1.25	20	141	168.25
9	150	1.25	30	395	378.0
10	200	2	30	218	216.0
11	150	1.25	30	106	102.75
12	100	1.25	40	126	115.75
13	150	2	20	387	390.25
14	150	0.5	20	287	259.75
15	150	2	40	165	182.0

**Table 4:** Analysis of variance (ANOVA) data for particle size (PS) obtained in various trials during the design of experiments by Box-Behnken design for the formulation of 2-methoxyestradiol-loaded polymeric micelles (2ME-PMs) using PEG-PLGA (PL), Tween 80 (TW80), and alpha-lipoic acid (ALA) as independent factors.

Source	Sum of Squares	Degrees of Freedom	Mean Square	F-Ratio	*p*-Value
A: PEG-PLGA (PL)	65,884.5	1	65,884.5	85.95	0.0002
B: Tween 80 (TW80)	16,200.0	1	16,200.0	21.14	0.0059
C: Alpha-lipoic acid (ALA)	22,472.0	1	22,472.0	29.32	0.0029
AA	1787.08	1	1787.08	2.33	0.1873
AB	36.0	1	36.0	0.05	0.8370
AC	8649.0	1	8649.0	11.28	0.0201
BB	1953.23	1	1953.23	2.55	0.1713
BC	4900.0	1	4900.0	6.39	0.0526
CC	132.923	1	132.923	0.17	0.6944
Total error	3832.5	5	766.5	---	---
Total (corr.)	126,157	14	---	---	---

**Table 5:** Optimization data suggested by the software by Box-Behnken design for the formulation of 2-methoxyestradiol-loaded polymeric micelles (2ME-PMs) using PEG-PLGA (PL), Tween 80 (TW80), and alpha-lipoic acid (ALA) as independent factors.

Factor	Low	High	Optimum
PEG-PLGA (PL) (mg)	100	200	100.282
Tween 80 (TW80) (%)	0.5	2.0	2.0
Alpha-lipoic acid (ALA) (mg)	20	40	40

## Data Availability

Not applicable.

## Supplementary Materials

The following are available online at https://www.mdpi.com/2073-4360/13/6/884/s1, Figure S1: HPLC representative spectra of 2ME.

## Abbreviations

2-ME, 2-methoxyestradiol; ALA, alpha-lipoic acid; COX-2, cyclooxygenase-2; IL-1β, interleukin-1β; IL-6, interleukin-6 (IL-6); NF-kB, nuclear factor kappa B; NO, nitric oxide; p53, tumor protein P53 (p53); PC, Prostate cancer; PEG, polyethylene glycol; PL, PEG-PLGA co-polymer; PLGA, poly(lactic-co-glycolic acid); PMs, polymeric micelles; polydispersity index; PS, particle size; PDI, RT-PCR, real-time polymerase chain reaction; TEM, transmission electron microscopy; TW80, Tween 80.
