# Supplementary material for: Development and In Vitro Evaluation of 2-Methoxyestradiol Loaded Polymeric Micelles for Enhancing Anticancer Activities in Prostate Cancer
Source: Polymers (Basel). 2021 Mar 13;13(6):884. doi: 10.3390/polym13060884 (PMC7998642; doi:10.3390/polym13060884)
Supplement: Supplementary file 1 [file polymers-13-00884-s001.pdf]

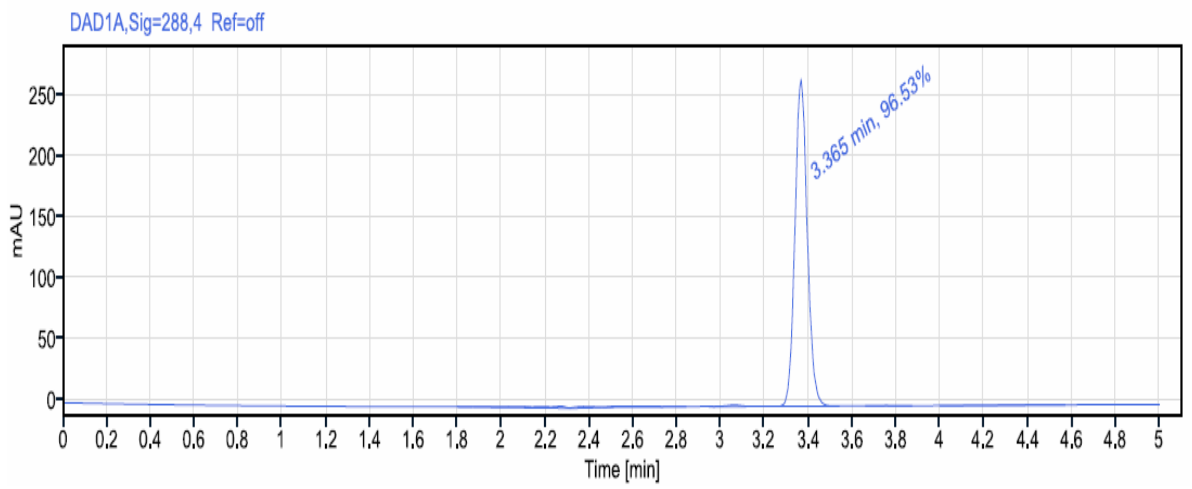

Figure S1. HPLC representative spectra of 2ME.

Highlights

- The PMs were optimized using a Box-Behnken design with the PL, TW80, and ALA as the independent variables and particle size (PS) as the response.
- The optimized PMs were satisfactory in terms of PS (65.36±2.2 nm), PDI (0.273±0.03), surface morphology, and entrapment efficiency (65.23±3.5%).
- The developed 2ME-PMs have been able to provide better cytotoxicity than 2ME alone.
- The formulation of 2ME-PMs could significantly improve the apoptotic activity compared to 2ME alone.
